# The Association between Air Pollution and Subclinical Atherosclerosis

**DOI:** 10.1289/ehp.1307403

**Published:** 2014-01-01

**Authors:** Tomoyuki Kawada

**Affiliations:** Department of Hygiene and Public Health, Nippon Medical School, Tokyo, Japan, E-mail: kawada@nms.ac.jp

Rivera et al. (2013) investigated the association between air pollution and subclinical atherosclerosis by using carotid intima media thickness (IMT), ankle–brachial index (ABI), and several indicators of air pollution. As a main outcome, air pollution was positively associated with an ABI of > 1.3, and also with changes in IMT. In contrast, they observed no significant association between air pollution and an ABI of < 0.9.

I have some concerns about their study (Rivera et al. 2013). First, the study included a small number of subjects with ABIs < 0.9 and > 1.3 (56 and 116, respectively). The authors used multinomial logistic regression analysis; for the full-adjustment model, > 16 air pollution variables were used. There is a limitation in the number of independent variables appropriate for multiple logistic regression analysis (Novikov et al. 2010; Peduzzi et al. 1996), and enough events should be included to maintain statistical power for multivariate analysis. According to the criteria that at least 10 events per variable are required to keep stable estimates (Peduzzi et al. 1996), Rivera et al. (2013) needed ≥ 170 events with an ABI < 0.9 or > 1.3 for their analysis.

Second, Rivera et al. (2013) used systolic and diastolic blood pressure to adjust for the relationship between air pollution and indicators of atherosclerosis. But multicollinearity among independent variables should have been considered in the analysis (York 2012).

Finally, Rivera et al. (2013) could not clarify the lack of association between ABI < 0.9 and indicators of air pollution. An ABI of 0.9–1.0 is also associated with cardiovascular risk (Ono et al. 2003). Therefore, the association between air pollution and subclinical atherosclerosis should be evaluated by selecting a higher cut-off value of ABI, such as 1.0. This procedure will increase the number of events for multivariate analysis.

Other researchers have reported a significant association between air pollution and IMT (Bauer et al. 2010; Diez Roux et al. 2008). Lenters et al. (2010) also examined the association between air pollutants and indicators of vascular damage but observed no association between air pollution and IMT. Lenters et al. (2010) used nitrogen dioxide (NO_2_), black smoke, particulate matter ≤ 2.5 μm in aerodynamic diameter (PM_2.5_), and sulfur dioxide (SO_2_) as indicators of air pollution, and they used pulse wave velocity and augmentation index in addition to IMT as indicators of vascular damage. Traffic intensity and proximity of residence to roads were also used as indicators of air pollution. Lenters et al. found significant associations only between NO_2_ and pulse wave velocity and augmentation index and between SO_2_ and pulse wave velocity. Because contradictory results for this association have been reported, further longitudinal studies are needed to assess this association.
